# Phenylalanine free infant formula in the dietary management of phenylketonuria

**DOI:** 10.1186/s13023-023-02621-9

**Published:** 2023-01-25

**Authors:** Ozlem Yilmaz, Barbara Cochrane, Jo Wildgoose, Alex Pinto, Sharon Evans, Anne Daly, Catherine Ashmore, Anita MacDonald

**Affiliations:** 1 Birmingham Women’s and Children’s Hospital, Birmingham, B4 6NH UK; 2 Department of Nutrition and Dietetics, Ankara Yildirim Beyazit University, 06760 Ankara, Turkey; 3 Dietetic Department, Royal Hospital for Children, Queen Elizabeth Hospital, Glasgow, 51 4TF UK; 4 Bradford Children’s Hospital, Bradford, BD5 0NA UK

**Keywords:** Phenylketonuria, Infancy, Infant protein substitute, Formula, Acceptability, Growth

## Abstract

**Background:**

Phenylalanine-free infant formula is an essential source of safe protein in a phenylalanine restricted diet, but its efficacy is rarely studied. We report a multicentre, open, longitudinal, prospective intervention study on a phenylalanine-free infant formula (PKU Start: Vitaflo International Ltd.).

**Results:**

This was a 2-part study: part I (28 days short term evaluation) and part II (12 months extension). Data was collected on infant blood phenylalanine concentrations, dietary intake, growth, and gastrointestinal tolerance. Ten infants (*n* = 8 males, 80%), with a median age of 14 weeks (range 4–36 weeks) were recruited from 3 treatment centres in the UK. Nine of ten infants completed the 28-day follow-up (one caregiver preferred the usual phenylalanine-free formula and discontinued the study formula after day 14) and 7/9 participated in study part II. The phenylalanine-free infant formula contributed a median of 57% (IQR 50–62%) energy and 53% (IQR 33–66%) of total protein intake from baseline to the end of the part II extension study. During the 12-month follow-up, infants maintained normal growth and satisfactory blood phenylalanine control. Any early gastrointestinal symptoms (constipation, colic, vomiting and poor feeding) improved with time.

**Conclusion:**

The study formula was well tolerated, helped maintain good metabolic control, and normal growth in infants with PKU. The long-term efficacy of phenylalanine-free infant formula should continue to be observed and monitored.

## Introduction

Phenylketonuria (PKU, OMIM 261600) is an autosomal recessive disorder caused by a deficiency of phenylalanine hydroxylase (PAH). Reduced PAH activity causes high phenylalanine concentrations in blood and tissues, and if untreated, results in microcephaly, severe developmental delay, epilepsy, and irreversible neurocognitive damage [1, 2]. It is identified by newborn screening, and it is recommended that treatment is initiated within the first 10 days of life in any infant with a blood phenylalanine > 360 µmol/L [2]. A monotherapy, a phenylalanine restricted diet, is commenced immediately, with the aim of supporting optimal neurocognitive performance, psychosocial functioning, growth and nutritional status [2, 3].

Dietary management necessitates the restriction of natural protein and supplementation with a low phenylalanine/phenylalanine-free protein substitute based on amino acids or glycomacropeptide. Protein substitutes provide essential and non-essential amino acids and commonly include micronutrients and essential fatty acids [2]. In infancy, at diagnosis, if blood phenylalanine levels are over 1000 µmol/l, any natural protein sources (human milk/standard infant formula) are temporarily stopped and replaced with a phenylalanine-free infant amino acid formula to ensure a rapid and immediate decrease in blood phenylalanine until therapeutic targets (120 to 360 µmol/L) are reached; this is usually within 2–3 days. A source of natural protein from human milk or standard infant formula is then introduced, with the volumes prescribed titrated with blood phenylalanine concentrations to maintain phenylalanine levels within the target range [2–4]. The phenylalanine-free infant formula will meet any deficit in energy and protein requirements that cannot be met by restricted volumes of human milk or standard infant formula. In infancy, protein substitutes provide 50–80% of the total protein requirements [2, 3]. Infants with classical PKU require a higher total protein intake than healthy populations due to inefficient absorption/utilization of amino acids from the protein substitute source [5, 6].

The nutritional composition of protein substitutes must comply with relevant worldwide regulations for infant formula. In the European Union (EU), the compositional requirements for infant formulas for special medical purposes must meet the Commission Delegated Regulation (EU) (2016/128) supplementing the Regulation (EU) No 609/2013 of the European Parliament and of the Council [7]. Phenylalanine-free infant amino acid formulas are now sophisticated in composition, being supplemented with vitamins, minerals, trace elements, and essential fatty acids [8, 9]. They aim to mimic the nutritional composition of human milk/standard infant formula (without phenylalanine) as closely as possible, and all raw materials used in their manufacture must be suitable for infants [8].

A better understanding of PKU has led to improved dietary practices in infant feeding [10]. In the 1950s special low phenylalanine infant protein substitute was commonly derived from casein hydrolysate. They contained some residual phenylalanine, supplemented with vitamins and minerals but were commonly deficient in one or more nutrients [11–14]. In 1970, a phenylalanine-free infant formula based on amino acids was developed [15]. It contained amino acids only, had a lower energy content, and improved acceptability compared with protein hydrolysates. It was supplemented with glucose and/or sugar, fat emulsion, minerals, and rose hip or blackcurrant juice with additional vitamins, making it complex to use in the early months of feeding. However, growth and blood phenylalanine control were satisfactory when evaluated in a small group of 5 infants [16]. Acosta et al. also reported the safe use of a nutritionally supplemented phenylalanine-free infant formula in a group of 35 infants over 6 months [17]. They grew adequately when consuming an average total protein intake of 2.9 g/kg during the first 3 months of life and 2.5 g/kg body weight between 4 and 6 months of age. In the last 50 years, there have been improvements in the nutritional composition of infant amino acid formulas designed for PKU [14]. Novel ingredients such as long chain polyunsaturated fatty acids [18] and prebiotics have been added to phenylalanine-free infant formula [8, 19], although the optimal formulation remains undefined [20, 21].

For more than 50 years, infants with PKU have been identified by newborn screening [22]. The prevalence of PKU varies worldwide, with an average of 1:23,930 newborns. It is highest in European and certain Middle Eastern countries. In Europe, the prevalence ranges widely, from 1:4000 live births in Italy and 1:4545 live births in Ireland to < 1:112,000 live births in Finland [23]. The average West Midlands, UK incidence of PKU is approximately 1:13,000 live births [24]. Until recently, only one suitable phenylalanine-free infant formula was available in the UK. Relying on one supplier leaves infants at significant risk if there is any interruption of formula supply due to issues with technical production, pandemics, reduced access to raw ingredients, or inefficient distribution systems. If the formula supply is disrupted, it is likely to have an immediate detrimental impact on blood phenylalanine control, clinical outcome, and nutritional intake in infants, triggering anxiety and distress in parents/caregivers and requiring extra and immediate health professional time and attention [2, 3].

Overall, clinical data on the use of phenylalanine-free amino acid-based infant formula is limited. We report a multicentre, open, longitudinal, prospective intervention study on a phenylalanine-free, amino acid-based infant formula for PKU (PKU Start: Vitaflo). We studied growth, metabolic control, and its acceptability and tolerability in a group of infants with PKU over 12 months of formula usage.

## Materials and methods

### Study design

**Fig. 1:**
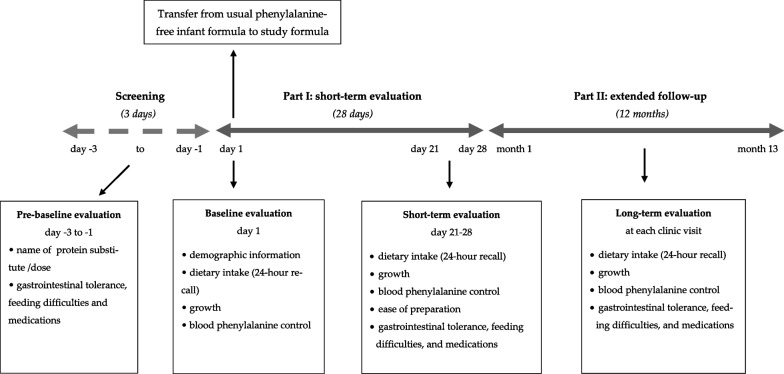
Schematic diagram of the study design. Part I: short-term evaluation; Part II: extended follow-up study in PKU infants using study formula

This was a 2-part study in full-term infants diagnosed with PKU and identified by newborn screening (Fig. 1). Infants were recruited from three specialist PKU centers in the UK: Birmingham Women’s and Children’s Hospital (*n *= 5), Royal Hospital for Children in Glasgow (*n *= 4), and Bradford Children’s Hospital (*n* = 1). Inclusion criteria included infants diagnosed with PKU by newborn screening and continuously treated with a low-protein diet supplemented with phenylalanine-free infant amino acid formula. Infants < 4 weeks or > 1 year of age, diagnosed with hyperphenylalaninaemia (blood phenylalanine concentrations < 360 µmol/L when untreated), or with a co-morbidity such as diabetes were excluded. Infants already established on solids were included in the study.

Part I: in a 28-day short-term acceptability and tolerance study, infants replaced their usual daily doses of infant amino acid formula (PKU Anamix Infant: Nutricia) with the same amount of study formula (PKU Start: Vitaflo). Subject demographics were recorded at baseline, including PKU classification according to pre-treatment blood phenylalanine levels (classical PKU [> 1200 µmol/L]), moderate PKU [600–1200 µmol/L], or mild PKU [360–600 µmol/L]), infant’s gestational age, sex, ethnicity, relevant medical history, and any concurrent medications. Information on the usual infant amino acid formula (dose, type, daily volume, and the number of feeds each day), source and amount of natural protein (breast milk or standard infant formula), and time of commencement of solid food intake were recorded from pre-baseline. Prior to baseline and during part I (28-day study), parents/caregivers completed daily questionnaires to record gastrointestinal tolerance/intolerance: occurrence of vomiting, regurgitation, abdominal discomfort or colic symptoms, flatulence, number of daily stools, and a description of their characteristics [loose, soft, hard], any feeding difficulties, and concomitant medications. The last three retrospective blood phenylalanine concentrations, weight, length, dietary energy, and total protein intake were collected at baseline and week 4. At the end of 4 weeks, parents described the ease of preparation and dissolving properties of the study formula.

Part II: an extended follow-up study was conducted with infants who continued to use the study formula for 12 months or until the study formula was discontinued. Dietary prescriptions (energy, total protein and natural protein intake, protein equivalent from protein substitute intake) and anthropometric measurements (weight and length) were documented by the dietitians at each clinic visit. Routine blood phenylalanine concentrations (twice weekly/weekly) were also recorded. Any relevant medications and gastrointestinal problems were reported by parents/caregivers.

### Study formula

The study formula (PKU Start, Vitaflo International Ltd.) was an amino acid-based powdered phenylalanine-free infant formula containing non-phenylalanine essential and non‐essential amino acids, carbohydrates, fat, vitamins, minerals, trace elements, arachidonic acid, and docosahexaenoic acid. It was reconstituted by adding 4.7 g of powder to 30 ml of water. It provided 2 g/100 ml of protein equivalent at a standard dilution of 14.1%. Table 1 shows the nutritional composition of the study formula compared to breast milk [25, 26] and an alternative powdered phenylalanine-free infant formula approved by the UK advisory board on borderline substances.

**Table 1:** Nutritional composition of the phenylalanine-free infant amino acid formula used in the study: comparison with pre-baseline phenylalanine-free infant amino acid formula, and breast milk

Nutritional Information (per 100 ml)	Units	PKU Start^a^ (Vitaflo)	PKU Anamix infant^b^ (Nutricia)	Mature breast milk^c^
Energy	kcal	68	70	70
	kj	287	293	291
Protein equivalent	g	2.0	2.0	1.07
Carbohydrate	g	7.1	7.5	7.4
Of which sugars	g	0.7	1.1	
Fat	G	3.5	3.5	4.2
Of which DHA	mg	14	17.8	13.6
Of which AA	mg	28	17.8	17.6
Vitamins				
Vitamin A	µg RE	64	61.2	60
Vitamin D	µg	1.6	1.7	0.01
Vitamin E	mg αTE	0.84	1.38	0.35
Vitamin C	mg	8.6	7.34	3.8
Vitamin K	µg	5.6	5.59	N/A
Thiamin	mg	0.06	0.08	0.01
Riboflavin	mg	0.07	0.08	0.03
Niacin	mg	0.46	0.35	0.23
Niacin equivalents	mg NE	1.1	0.57	0.62
Vitamin B6	mg	0.06	0.08	0.06
Folic acid	µg	10.8	8.25	5.2
Vitamin B12	µg	0.17	0.18	0.01
Biotin	µg	2.7	2.7	0.8
Pantothenic acid	mg	0.35	0.42	0.26
Choline	mg	21.3	21.9	N/A
Minerals				
Sodium	mg	27	28.7	15
Potassium	mg	71	75.8	60
Chloride	mg	51	53.3	43
Calcium	mg	56	61.5	35
Phosphorus	mg	42	45	15
Magnesium	mg	6.3	8.8	2.8
Trace elements				
Iron	mg	0.81	1.19	0.07
Copper	µg	56	63	39
Zinc	mg	0.49	0.84	0.3
Manganese	mg	0.04	0.004	N/A
Iodine	µg	14.8	14.7	7
Molybdenum	µg	2.2	1.82	N/A
Selenium	µg	3.0	2.7	1.4
Chromium	µg	1.8	2.1	N/A
Amino acids				
Alanine	g	0.08	0.09	0.05
Arginine	g	0.14	0.16	0.05
Aspartic acid	g	0.22	0.15	N/A
Cystine	g	0.05	0.06	0.03
Glutamine	g	0.16	0.21	0.23
Glycine	g	0.21	0.14	0.03
Histidine	g	0.08	0.09	0.03
Isoleucine	g	0.15	0.14	0.07
Leucine	g	0.23	0.24	0.12
Lysine	g	0.15	0.17	0.09
Methionine	g	0.04	0.04	0.02
Phenylalanine	g	0.00	0.00	0.05
Proline	g	0.15	0.17	0.12
Serine	g	0.10	0.11	0.05
Threonine	g	0.15	0.12	0.05
Tryptophan	g	0.05	0.05	0.03
Tyrosine	g	0.22	0.22	0.04
Valine	g	0.17	0.16	0.09

PKU, phenylketonuria; DHA, docosahexaenoic acid; AA, arachidonic acid; N/A, not applicable

^a^At standard dilution of 14.1%

^b^At standard dilution of 15.0%

^c^Values are from Oppe, 1977. DHA and AA contents were calculated from Giuffrida et al. (2022)

### Stool characteristics, gastrointestinal symptoms and medications

Data on gastrointestinal symptoms (constipation, abdominal discomfort, colic, vomiting, regurgitation, flatulence), any feeding difficulties, and concomitant medications were collected for three days pre-baseline, 28-day in the short-term evaluation period, and at each clinic visit during the 12-month follow-up. Parents/caregivers recorded stool characteristics (three categories: soft, loose, and hard) pre-baseline and 28-day in the short-term evaluation period.

### Anthropometry

Length was measured using a Holtain Harpenden infantometer (Holtain Ltd., Crymych, UK) and weight on calibrated digital baby scales (Seca, Medical Measuring Systems and Scales, Model 875, UK). Weight was measured to the nearest 0.1 g and length to the nearest 0.1 cm. Data were converted to age-based z-scores for weight, length, and body mass index (BMI) according to WHO/UK growth definitions [27, 28].

### Blood phenylalanine levels

Weekly/twice weekly morning fasting heel prick blood spots for phenylalanine were collected on filter cards, Perkin Elmer 226 (UK Standard NBS) by caregivers at home. The number of fasting hours depended on the number of infant night feeds and the interval between feeds. All caregivers had received blood spot training from a specialist nurse. All blood spot samples were sent by first class post to the hospital laboratorities for phenylalanine analysis. The cards had a standard thickness, and the blood phenylalanine concentrations were calculated on a 3.2 mm punch by MS/MS tandem mass spectrometry.

### Dietary assessment

Dietary intake was determined by a 24-h recall recorded at baseline, week 4, and at each clinic visit during a 12-month long-term follow-up. Prescribed energy, total protein, natural protein, and protein equivalent from protein substitutes were assessed using the computer software nutritional analysis program Nutritics [29].

### Statistical analysis

Sample size calculations were not performed due to the rarity of the condition and the exploratory nature of the study. Only descriptive statistics were used to present the results of this study.

### Ethical approval

The study was approved by the Northwest Liverpool East Research Ethics Committee and granted a favourable ethical opinion, reference number 19/LO/1027 and IRAS (Integrated Research Application System) 265417. Written informed consent was obtained for all subjects from at least one caregiver with parental responsibility.

## Results

### Subjects

Ten full-term infants (*n *= 8 males, 80%), with a median age of 14 weeks (range 4–36 weeks), were recruited (Table 2). Nine infants (90%) were Caucasian, and 1 (10%) was of Pakistani Asian origin. Ninety percent (*n *= 9) of infants completed the first 28-day follow-up, and *n *= 1 withdrew on day 14. Two infants who completed the short-term evaluation (part I) withdrew from the long-term follow-up (part II). Seven infants participated in study part II, one infant stopped the study formula at 10 months follow-up, and six completed the full 12 months extension study.

**Table 2:** Demographic informations and clinical characteristics of subjects

Subject number	Sex	Age at baseline (weeks)	Ethnicity	PKU classification ^a^	% (Number) of days completed in Part I	% (Number) of months completed in Part II	Protein substitute profile at pre-baseline					Source of natural protein	Commencement of solid intake
							Number of different protein substitutes/day	Type of protein substitutes	Number of doses/day	Total protein equivalent from all protein substitutes (g/day)	Protein equivalent from Phe-free infant formula ^b^ g/day (%)		
1	M	32	Caucasian	Mild	50% (14)	Withdrawn	2	Infant powder and weaning semi-solid	3	20	5 (25%)	Standard infant formula / solid food	26 weeks
2	M	36	Caucasian	Classical	100% (28)	100% (12)	2	Infant powder and weaning semi-solid	3	29	10 (35%)	Solid food	16 weeks
3	M	8	Caucasian	Classical	100% (28)	Withdrawn	1	Infant powder	6	5	5 (100%)	Breast milk	Not applicable
4	F	6	Caucasian	Classical	100% (28)	100% (12)	1	Infant powder	5	6	6 (100%)	Standard infant formula	Not applicable
5	M	10	Pakistani Asian	Mild	100% (28)	100% (12)	1	Infant powder	7	8	8 (100%)	Standard infant formula	Not applicable
6	F	32	Caucasian	Classical	100% (28)	Withdrawn	2	Infant powder and weaning semi-solid	3	20	10 (50%)	Standard infant formula / solid food	17 weeks
7	M	18	Caucasian	Classical	100% (28)	100% (12)	1	Infant powder	6	14	14 (100%)	Standard infant formula	Not applicable
8	M	17	Caucasian	Classical	100% (28)	100% (12)	1	Infant powder	4	10	10 (100%)	Standard infant formula	Not applicable
9	M	4	Caucasian	Classical	100% (28)	100% (12)	1	Infant powder	5	9	9 (100%)	Standard infant formula	Not applicable
10	M	6	Caucasian	Classical	100% (28)	83% (10)	1	Infant powder	6	14	14 (100%)	Standard infant formula	Not applicable

Part I: short-term evaluation over 28 days. Part II: extended follow-up for 12 months

PKU, phenylketonuria; M, male; F, female

^a^Mild PKU with pre-treatment phenylalanine levels of 360–600 μmol/L, and classical PKU with pre-treatment phenylalanine > 1200 μmol/L

^b^Protein equivalent intake (g/day) from the pre-baseline phenylalanine-free infant formula (PKU Anamix infant, Nutricia)

At pre-baseline, all infants (*n *= 10) took PKU Anamix infant (Nutricia) as their usual infant protein substitute. The median (range) intake of protein equivalent from the usual infant protein substitute was 10 g/day (range 5–14 g/day). Three infants (30%) had already started solid foods with an additional weaning protein substitute (PKU Anamix First Spoon *n *= 2; PKU Gel *n *= 1) when they were aged between 16 to 26 weeks. The phenylalanine source was either standard infant formula (*n *= 6, 60%), a combination of standard infant formula and solid food (*n *= 2, 20%), human breast milk directly from the breast (*n *= 1, 10%), or solids only (*n *= 1, 10%), determined by infant age.

### Subject withdrawal

At baseline, all infants (*n *= 10) changed all their phenylalanine-free infant formula requirements to the study formula PKU Start (Vitaflo International) without difficulty. Caregivers of one infant (Subject 1) preferred his usual phenylalanine-free infant formula and discontinued the study formula after 14 days, although there had been no tolerance issues with the study formula. Two infants did not complete the extension study; in one subject, the caregivers preferred not to progress any further with the study formula as they could not see any advantage over their usual infant phenylalanine-free infant formula and had large supplies of the latter (Subject 3), and in one centre, one subject was not followed up due to dietetic changes within the department (Subject 6). One infant (Subject 10) completed 10 months of the extension study because he stopped phenylalanine-free infant formula at one year of age and he had fully transitioned to a weaning protein substitute (PKU Gel).

### Gastrointestinal symptoms, feeding issues and medications

Gastrointestinal symptoms, feeding issues, and any relevant medications at pre-baseline, during the 28-day evaluation (Part I), and long-term follow-up (Part II) are given in Table 3. Pre-baseline: *n *= 7 (70%) infants had constipation, *n *= 5 (50%) colic, and *n *= 3 (30%) vomiting. All gastrointestinal symptoms improved over time, and constipation was not reported long-term. Throughout the long-term follow-up, *n *= 2 (29%) experienced vomiting: in one infant, this was attributed to viral infection, and the second infant had a long history of vomiting, mostly post-feeding. Loose stools were more common at baseline (*n *= 4, 40%) than when taking the study formula (*n *= 1, %10). Pre-baseline: *n *= 2 (20%) infants were prescribed laxatives, *n *= 2 (29%) anti-colic medication, and *n *= 1 antacids. In the 12-month follow-up, no infants were prescribed medications, except *n *= 1, who was given antacids due to persistent vomiting.

**Table 3:** Gastrointestinal symptoms, feeding issues and medications at pre-baseline, during the short-term evaluation (28 days) and extended follow-up (12 months)

	Pre-baseline (*n* = 10)	Short-term ^a^ (28 days, *n* = 10)	Long-term (12 months, *n* = 7)
Gastrointestinal symptoms (*n*, %)			
Constipation	7 (70%)	6 (60%)	0 (0%)
Colic	5 (50%)	3 (30%)	1 (14%)
Vomiting	3 (30%)	3 (30%)	2 (29%)
Stool consistency (*n*, %)			
Soft	6 (60%)	8 (80%)	N/A
Loose	4 (40%)	1 (10%)	N/A
Hard	0 (0%)	1 (10%)	N/A
Poor feeding (*n*, %)	5 (50%)	3 (30%)	1 (14%)
Medications (*n*, %)			
Laxatives	2 (20%)	2 (20%)	0 (0%)
Colic treatment	2 (20%)	1 (10%)	0 (0%)
Antacids/antiemetics	1 (10%)	1 (10%)	1 (14%)

N/A, does not apply

^a^One infant who discontinued the study formula at day 14 (subject 1) had a daily diary during the first 14-day and was included in the analysis

### Blood phenylalanine control

**Fig. 2:**
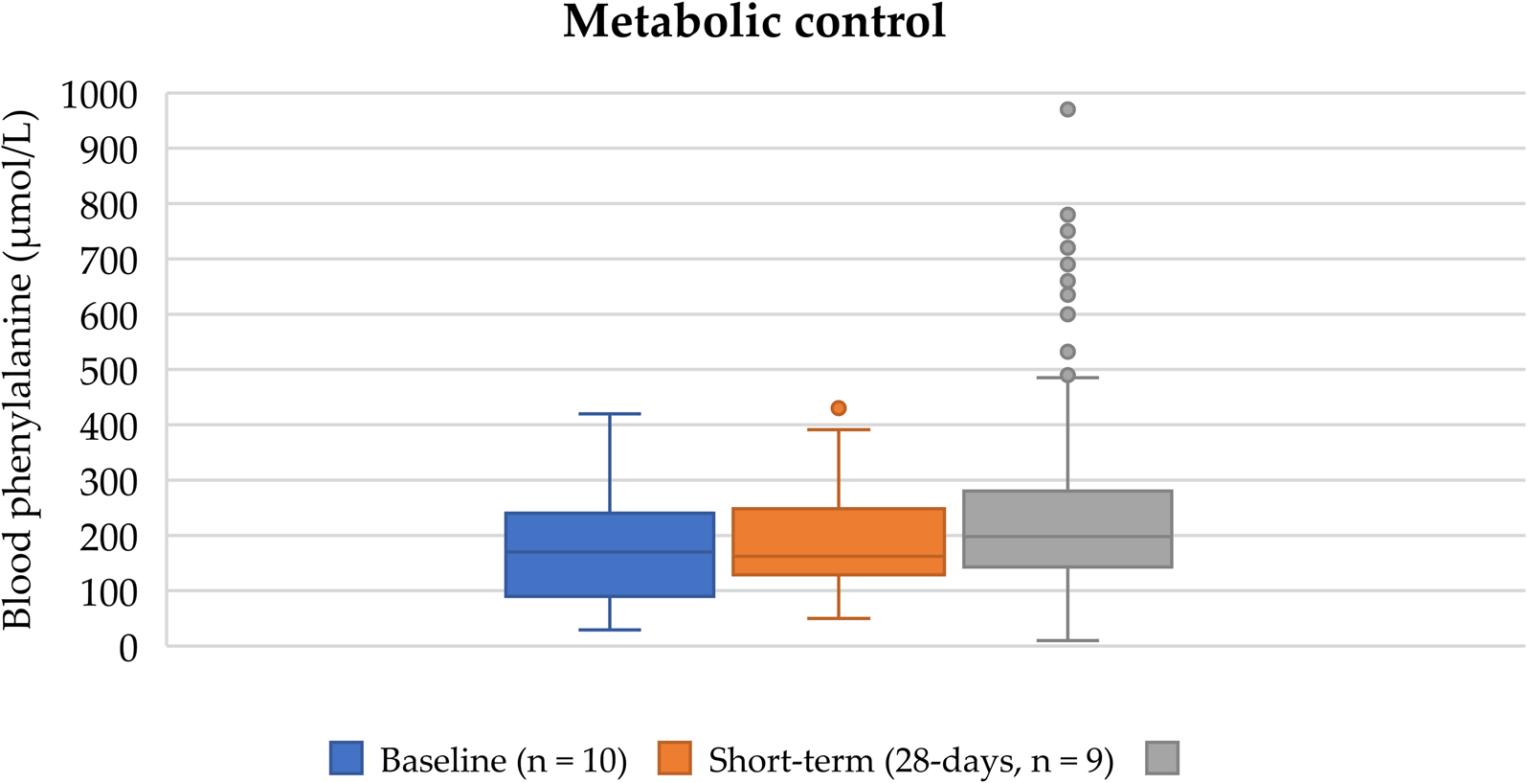
Blood phenylalanine control of infants during baseline, short-term evaluation, and extended follow-up when taking the study phenylalanine-free infant formula

Median blood phenylalanine concentrations were within the therapeutic treatment target range of 120–360 µmol/L [2] during the study period: 170 µmol/L (IQR 91–225 µmol/L) at baseline, 162 µmol/L (IQR 130–245 µmol/L) at study week 4, and 198 µmol/L (IQR 144–280 µmol/L) in the 12-month extension study (Fig. 2). Over the study period, 90% of blood phenylalanine levels were below the upper target range of 360 µmol/L. During the 12-month extension study, any high blood phenylalanine levels were usually associated with infections, vomiting or diarrhoea when infant study formula was refused or not tolerated.

### Changes in anthropometric characteristics

**Fig. 3:**
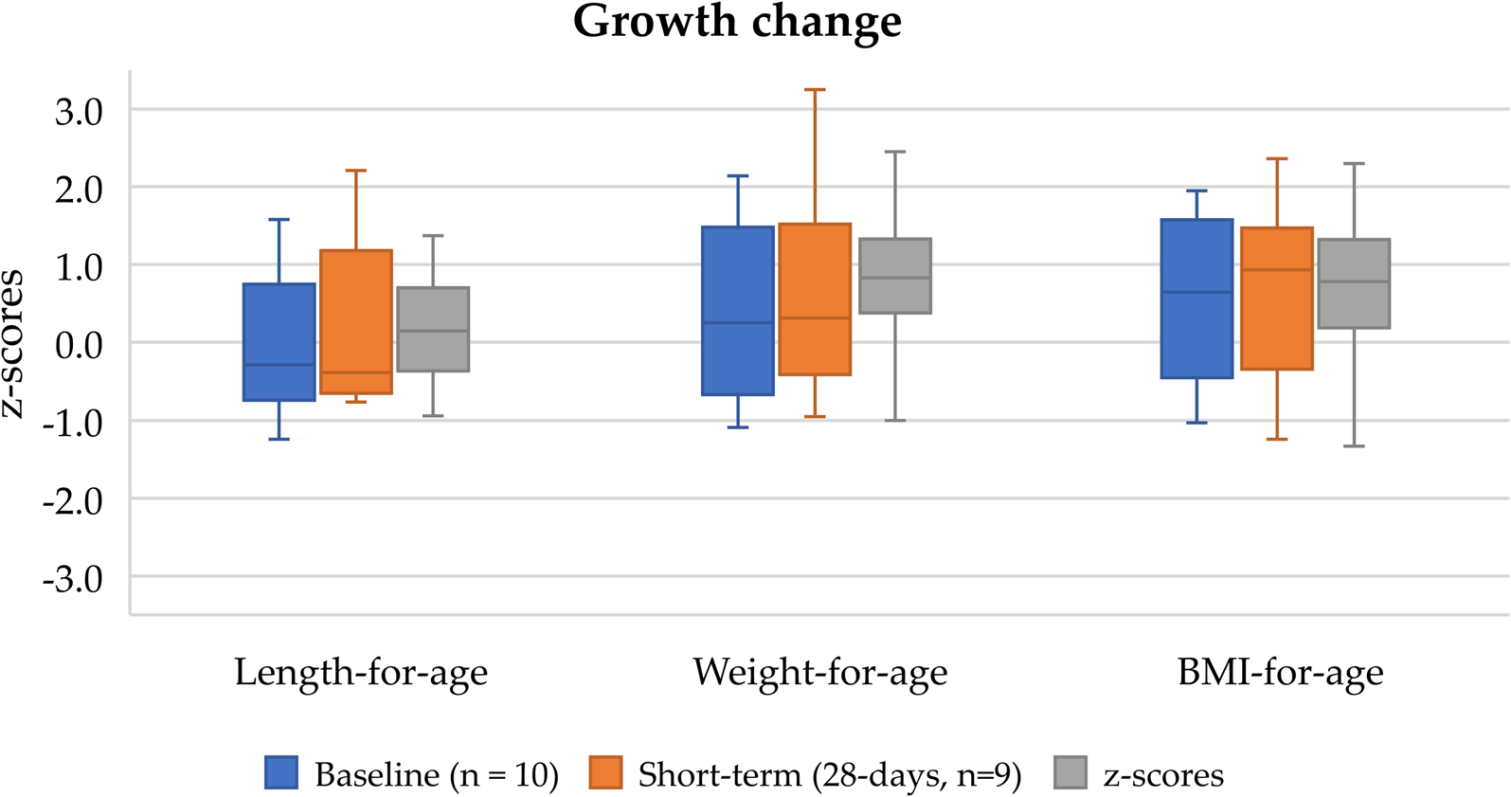
Change in median height-for-age, weight-for-age, and BMI-for-age z-scores from baseline to the long term extended follow-up in infants using the study phenylalanine-free infant formula

Figure 3 shows the changes in median height-for-age, weight-for-age, and BMI-for-age z-scores. The median number of anthropometric measurements during the long-term evaluation was 5 (range 4–7).

Length for age: median z-score was − 0.3 (IQR − 0.7 to 0.6) at baseline (*n *= 10) and − 0.4 (IQR − 0.6 to 0.8) at week 4 (*n *= 9). Length-for-age z-score improved over time and was a median of 0.1 (IQR − 0.3 to 0.7) in the 12-month extension study (*n *= 7).

Weight for age: median z-score was 0.3 (IQR − 0.6 to 1.3) at baseline (*n *= 10), 0.3 (IQR − 0.4 to 1.3) at week 4 (*n *= 9), and 0.8 (IQR 0.4–1.3) in the 12-month extension study (*n *= 7).

BMI for age: median z-score was 0.6 (IQR − 0.3 to 1.4) at baseline (*n *= 10), 0.9 (IQR 0.2–1.3) at week 4 (*n *= 9), and 0.8 (IQR 0.2–1.3) in the 12-month extension study (*n *= 7).

### Dietary intake

Table 4 shows the dietary intake of infants at baseline (n = 9), at week 4 (n = 8), and each clinic visit during the long-term evaluation (n = 7). The median number of dietary assessments during the long-term evaluation was 6 (range 3–8). The median energy intake as a percentage of the estimated average energy requirements (EAR) was 110% (IQR 91–115) at baseline, 103% (IQR 85–110%) at week 4, and 90% (IQR 79–101%) in the 12-month extension study.

**Table 4:** Median energy, total protein, natural protein, and protein equivalent intake from phenylalanine-free infant amino acid formula

	Energy intake				Protein intake						
	EAR %	kcal/kg/d	kcal/d	% kcal from the study formula	Total (g/d)	Total (g/kg/d)	PE (g/d) from Phe-free infant formula ^b^	PE (g/kg/d) from Phe-free infant formula^b^	% From Phe-free infant formula	Natural (g/d)	Natural (g/kg/d)
Baseline (*n* = 9)^a^	110 (91–115)	104 (92–111)	618 (547–746)	50 (45–57)	18 (14–31)	3.1 (2.7–3.5)	11 (8–13)	1.3 (1.2–1.9)	55 (34–62)	5.9 (5.9–6.0)	0.8 (0.6–1.0)
Short-term (28-day) Evaluation (*n* = 8)^a^	103 (85–110)	91 (81–101)	662 (632–803)	54 (51–65)	18 (16–24)	2.5 (2.3–2.7)	12 (9–15)	1.5 (1.3–1.7)	62 (58–74)	5.3 (4.4–5.9)	0.6 (0.5–0.8)
Extension study (12-month) follow-up (*n* = 7)	90 (79–101)	84 (68–96)	662 (620- 797)	59 (51–62)	21 (17–31)	2.5 (2.0–3.2)	11 (8–12)	1.0 (0.6–1.4)	49 (31–65)	4.8 (4.5–5.5)	0.5 (0.4–0.6)
Mean (*n* = 9)	94 (81–109)	90 (74–101)	662 (605–792)	57 (50–62)	19 (17–31)	2.6 (2.1–3.2)	11 (8–13)	1.2 (0.8–1.6)	53 (33–66)	5.1 (4.5–5.9)	0.6 (0.4–0.7)

EAR, estimated average requirements; Phe, phenylalanine; PE, protein equivalent

^a^One breastfed infant was not included

^b^Protein equivalent intake from phenylalanine-free infant amino acid formula only (excluding weaning protein substitute)

The median total protein intake (including natural protein and protein equivalent from study phenylalanine-free infant formula and weaning protein substitutes) decreased from 3.1 g/kg/day (IQR 2.7–3.5 g/kg/day) at baseline to 2.5 g/kg/day (IQR 2.3–2.7 g/kg/day) at week 4 and remained consistent at a median of 2.5 g/kg/day (IQR 2.0–3.2 g/kg/day) in the 12-month extension study. The study phenylalanine-free infant formula provided a median of 57% (IQR 50–62%) of energy and 53% (IQR 33–66%) of total protein intake from baseline to the end of the 12-month extension study. The median protein equivalent intake from the phenylalanine-free infant study formula remained relatively constant at 11–12 g/day throughout the study period. When expressed as g/kg/day, the median protein equivalent intake from the study phenylalanine-free infant formula decreased from 1.5 g/kg/day (IQR 1.3–1.7 g/kg/day) at week 4–1.0 g/kg/day (IQR 0.6–1.4 g/kg/day) in the 12-month extension study. The median natural protein intake remained stable at a median of 0.6–0.8 g/kg/day at baseline and week 4, then decreased to 0.5 g/kg/day in the 12-month extension study.

### Ease of formula preparation

Parents gave opinion on the ease of preparation and the dissolving properties of the study formula at the end of the 28-day. There was no difference in ease of preparation between the two infant formulas. Six parents/caregivers reported that the study formula mixed easily, and *n *= 4 reported it was satisfactory, with two describing that the study formula required hot water or more shaking to dissolve it. Two parents/caregivers said it was easier to clean the infant bottles because the study formula left less residue/film/grease around the bottle.

## Discussion

In PKU, it is important to evaluate and report the growth, acceptance, and tolerance of any new phenylalanine-free infant formula, to enable national policymakers to assess its efficacy. This is the first long-term, multi-centre study to evaluate the gastrointestinal tolerance, growth, and metabolic control of a new phenylalanine-free infant amino acid-based formula (PKU Start) designed for infants with PKU. The study formula was well accepted and tolerated by 90% (*n *= 9) of infants in a 28-day study and most infants continued with the study formula longer term. During the 12-month follow-up, infants maintained adequate/normal growth and clinically acceptable blood phenylalanine control. Any early gastrointestinal symptoms (constipation, colic, vomiting) improved with time. The phenylalanine-free infant formula contributed a median of 57% (IQR 50–62%) of energy intake and 53% (IQR 33–66%) of total protein intake from baseline to the end of the 12-month extension study.

The World Health Organization (WHO) advocates exclusive breastfeeding for the first 6 months of life [30], but in PKU, breast milk intake must be limited to maintain target therapeutic blood phenylalanine concentrations (120–360 μmol/L) [3, 31, 32]. Therefore, an amino acid infant formula is necessary to provide the remaining non-phenylalanine protein and nutrient requirements [33]. Although the production of an identical product to human milk is not feasible, infant formula attempts to mimic the nutritional composition of breast milk [34]. Human breast milk is a complex matrix with a general composition of 7% lactose, 1% protein, and 4% fat. Fat and lactose, contribute 54% and 42% of the total energy of breast milk, respectively. The study formula contained a similar carbohydrate (7%) and fat (3.5%) but higher protein (2%) content compared to human breast milk (Table 1).

Lactose is the primary carbohydrate in human milk and provides the primary energy source for the infant, improving health by modulating gut physiology, including prebiotic effects, stool softening, and effective absorption of water, calcium, and sodium [35, 36]. Although lactose has several advantages, alternative sources of carbohydrates such as pre-cooked starch, gelatinized starch, and maltodextrin are now commonly used in infant formula [37]. The carbohydrate source of the study formula was maltodextrin and glucose syrup. Maltodextrin and glucose have a similar energy value (4 kcal/g) to lactose but differ in digestion and absorption. Glucose polymers have a higher glycaemic index (GI = 110) than lactose (GI = 46), resulting in a higher glycaemic response [38]. The exclusion of lactose in nonallergenic infant formulas is due to potential contamination with milk proteins [39], and a specific need for lactose in phenylalanine-free infant formula in infants with PKU has not been studied. Therefore, the long-term consequences of replacing lactose with alternative carbohydrate sources in infant formula for health and biological functioning require investigation.

Fats are a crucial component of breast milk, supplying energy and aiding the development of the central nervous system [34, 40]. Triglycerides are the main lipid fractions in breast milk, accounting for approximately 95% of lipids, of which about 40% are saturated fatty acids (primarily palmitic acid) and 36% are monounsaturated fatty acids [35, 41]. Most infant formulas use vegetable oils derived from the palm (kernel/olein) oil, sunflower oil, coconut oil, rapeseed oil, and safflower oil to mimic the fatty acid profile of breast milk [40, 42, 43]. Palm oil may reduce fat and calcium absorption, contribute to stool hardness, and negatively impact health [44]. Fats in the study formula included the following vegetable oils, high oleic sunflower oil, coconut oil, canola oil, sunflower oil but no palm oil. Independent of the fat source used in infant formulas, The Commission Delegated Regulation (EU) 2016/127 mandates the addition of 20–50 mg docosahexaenoic acid/100 kcal for regular infant formula, while the addition of arachidonic acid remains optional [7]. The formula in the present study contained 21 mg/100 kcal docosahexaenoic acid and 41 mg/100 kcal arachidonic acid from fish oil and was within the range of the relevant European standards [7].

The European PKU guidelines advocate an additional 40% more protein from phenylalanine-free amino acids than the FAO/WHO/UNU safe levels [45] to account for the poor utilization of L-amino acids, ineffective absorption of natural/intact protein, and to aid the lowering of blood phenylalanine concentrations [2, 3, 14]. Reliance on synthetic nitrogen sources may compromise growth, but studies are conflicting. Earlier studies report an association between higher protein intake (natural protein, protein equivalent from protein substitutes, or both) and better growth outcomes in the early years of life [17, 46–48]. In contrast, other authors showed no relationship between growth and protein intake [49–51]. In a recent case-control study from our centre [52], infants aged 0–2 years had normal growth comparable to controls without PKU. Children in both groups exceeded the safe levels of protein intake (mean PKU 194%, range 141–251%; mean control 188%, range 133–272%) [2]. Similarly, in our current study, total protein intake (≥ 2.5 g/kg/day) exceeded the European PKU Guideline recommendation [2], and infants maintained normal height, weight, and BMI z-scores. Evidence suggests that a high protein intake in early life increases the stimulation of insulin and insulin growth factor 1 (IGF-1) leading to rapid weight gain [53]. The European Childhood Obesity Trial [54] has described increased total and free IGF-I in infants using higher protein formula (containing 2.9 and 4.4 g protein/100 kcal). A study in infant monkeys [55] showed that the higher protein content of standard infant formula increases appetite and calorie intake, suggesting a lack of ability to self-regulate energy intake, although we did not observe infants consume a high energy intake associated with increased volumes of phenylalanine-free infant formula. The long-term impact of the protein and free amino acids in infant formula on growth in PKU remains to be investigated.

Protein substitutes typically contain a good proportion of non-phenylalanine large neutral amino acids (LNAA), such as tyrosine, tryptophan, threonine, methionine, valine, isoleucine, leucine, and histidine, and the study formula provided 17% more LNAA than human milk (LNAA mg/g protein: study formula, 545 and human milk, 467) [56, 57]. This is in order to optimize neurotransmitter and protein synthesis while supporting physiological absorption [58]. LNAAs have the ability to block phenylalanine transport into the brain [59] and increase cerebral LNAA and neurotransmitter concentrations because they use the same transport system LNAA transporter LAT1 [56]. More studies are needed to define the optimal amino acid composition of phenylalanine-free infant formula.

The study shows that the study formula was well tolerated, with no adverse reactions, such as gastrointestinal intolerance occurring over the 12-month extension study. Although little is known about the gastrointestinal function of infants with amino acid disorders, MacDonald et al. [59] reported that caregivers of pre-school children with PKU perceived them to have more gastrointestinal issues, possibly due to the consumption of high osmolar protein substitutes. The osmolality of the study amino acid infant formula (350 mOsm/kg) was comparable to that of standard infant formula (< 400 mOsm/kg) [60], the baseline amino acid-based infant formula (380 mOsm/kg), and breast milk (300 mOsm/kg) [61]. It has been recommended that the osmolality of the infants’ feed should not exceed 450 mOsm/kg in infants with normal gastrointestinal function [62]. A systematic review showed no association between the osmolality of the infant feeds in the range of 300–500 mOsm/kg and adverse gastrointestinal symptoms [61]. Although gastrointestinal symptoms were common at baseline, constipation resolved in 7 infants, and no infants were prescribed long-term laxatives or anti-colic medications, except one infant who was given antacids due to persistent vomiting while taking the study formula. However, gastrointestinal symptoms are very common, especially during the first 6 months of life [63], and the improvement of gastrointestinal symptoms could be related to increased dietary diversity and/or increasing age.

Several novel ingredients in infant formulas have been introduced [14, 20], including prebiotics. MacDonald et al. [8] demonstrated that phenylalanine-free infant formula supplemented with galactooligosaccharides and fructooligosaccharides helped maintain levels of bifidobacteria and lower stool pH in infants with PKU, which may be associated with a reduced risk of infection [8]. This study formula did not contain prebiotics, and the effects of phenylalanine-free infant formula on gut-microbiome composition warrants further investigation. In recent years, glycomacropeptide (cGMP) derived from whey protein and supplemented with amino acids has been introduced as an alternative for phenylalanine-free protein substitutes [64] for patients from the age of 4 years with PKU. cGMP has been associated with prebiotic, antimicrobial, anti-cariogenic, gastric acid inhibitor, appetite control, and immune-modulatory activities [65], suggesting that it might be a good candidate for use as an alternative phenylalanine-free infant formula; however, this type of formula has not been developed for infants with PKU.

This study had a number of limitations. This was an exploratory, uncontrolled open study with small sample size and no crossover design. In general, it is challenging to enroll high numbers of infants with PKU in studies due to the rarity of conditions and caregiver reluctance to participate due to fear of moving to experimental infant formula at a time when they are still adjusting to the diagnosis. Also the number of national PKU centres in a position to conduct the infant formula studies is limited. The pre-baseline data was only collected over three days so may not accurately reflect gastrointestinal symptoms and any feeding difficulties of infants on their usual amino acid formula. Dietary assessments were collected by 3 dietitians from 3 metabolic centres so that they may have interpreted intake differently. However, the dietitians were very experienced, reviewed patients regularly, and had a good knowledge of their patients’ dietary intakes and patterns. In addition, the infant formula and food intake were regulated and varied little from day to day. Some of the infants received complementary feeding and were taking a second-stage protein substitute, which may have reduced the strength of our findings. No nutritional biochemistry or quantitative plasma amino acid data was collected.

## Conclusion

The study formula was well tolerated, maintained good metabolic control and normal growth, and was safe for use in the dietary treatment of PKU infants. Increased availability of phenylalanine-free infant formula alternatives ensures product availability in the event of a supply failure. Further studies involving larger cohorts of patients should be conducted to examine growth, metabolic control, and gastrointestinal tolerance on phenylalanine-free infant formulas.

## Data Availability

The data presented in this study are available on request from the corresponding author

## Abbreviations

PKU: Phenylketonuria
PAH: Phenylalanine hydroxylase
BMI: Body mass index
